# Publisher Correction: Epigenetic alterations of repeated relapses in patient-matched childhood ependymomas

**DOI:** 10.1038/s41467-022-35539-0

**Published:** 2022-12-22

**Authors:** Sibo Zhao, Jia Li, Huiyuan Zhang, Lin Qi, Yuchen Du, Mari Kogiso, Frank K. Braun, Sophie Xiao, Yulun Huang, Jianfang Li, Wan-Yee Teo, Holly Lindsay, Patricia Baxter, Jack M. F. Su, Adekunle Adesina, Miklós Laczik, Paola Genevini, Anne-Clemence Veillard, Sol Schvartzman, Geoffrey Berguet, Shi-Rong Ding, Liping Du, Clifford Stephan, Jianhua Yang, Peter J. A. Davies, Xinyan Lu, Murali Chintagumpala, Donald William Parsons, Laszlo Perlaky, Yun-Fei Xia, Tsz-Kwong Man, Yun Huang, Deqiang Sun, Xiao-Nan Li

**Affiliations:** 1 Pre-clinical Neuro-oncology Research Program, Texas Children’s Hospital, Baylor College of Medicine, Houston, TX 77030 USA; 2 Texas Children’s Cancer Center, Texas Children’s Hospital, Baylor College of Medicine, Houston, TX 77030 USA; 3 Jane and John Justin Neurosciences Center, Cook Children’s Medical Center, Fort Worth, TX 76104 USA; 4 Hematology and Oncology Center, Cook Children’s Medical Center, Fort Worth, TX 76104 USA; 5 Center for Epigenetics & Disease Prevention, Texas A&M University, Houston, TX 77030 USA; 6 Center for Translational Cancer Research, Institute of Biosciences and Technology, Texas A&M University, Houston, TX 77030 USA; 7 State Key Laboratory of Respiratory Disease, National Clinical Research Center for Respiratory Disease, Guangzhou Institute of Respiratory Health, the First Affiliated Hospital of Guangzhou Medical University; and Guangzhou Laboratory, Bioland, 510120 Guangzhou, Guangdong P. R. China; 8 Program of Precision Medicine PDOX Modeling of Pediatric Tumors, Division of Hematology-Oncology, Neuro-Oncology & Stem Cell transplantation, Ann & Robert H. Lurie Children’s Hospital of Chicago; Department of Pediatrics, Northwestern University Feinberg School of Medicine, Chicago, IL 60611 USA; 9 Department of Neurosurgery and Brain and Nerve Research Laboratory, the First Affiliated Hospital, and Department of Neurosurgery, Dushu Lake Hospital, Suzhou Medical College, Soochow University, 215007 Suzhou, P. R. China; 10 Humphrey Oei Institute of Cancer Research, National Cancer Center Singapore, Singapore, 169610 Singapore; 11 Cancer and Stem Cell Biology Program, Duke-NUS Medical School Singapore, Singapore, Singapore; 12 KK Women’s & Children’s Hospital Singapore, Singapore, Singapore; 13 Institute of Molecular and Cell Biology, A*STAR, Singapore, Singapore; 14 Department of Pathology, Texas Children’s Hospital, Baylor College of Medicine, Houston, TX 77030 USA; 15 Epigenetic Services, Diagenode, Liège Belgium; 16 State Key Laboratory of Oncology in South China, Collaborative Innovation Center for Cancer Medicine; Department of Radiation, Sun Yat-sen University Cancer Center, 510060 Guangzhou, Guangdong P. R. China; 17 Clinical Cytogenetic Laboratory, Department of Pathology, Northwestern University Feinberg School of Medicine, Chicago, IL 60611 USA

**Keywords:** CNS cancer, Paediatric cancer, DNA methylation

Correction to: *Nature Communications* 10.1038/s41467-022-34514-z, published online 05 November 2022

The original version of this Article omitted listing Dr. Deqiang Sun as a corresponding author. This has now been corrected in both the PDF and HTML versions of the Article.

