# Supplementary material for: Epigenetic Alterations of Repeated Relapses in Patient-matched Childhood Ependymomas
Source: Nat Commun. 2022 Nov 5;13:6689. doi: 10.1038/s41467-022-34514-z (PMC9637194; doi:10.1038/s41467-022-34514-z)
Supplement: Supplementary file 1 — Supplementary Information [file 41467_2022_34514_MOESM1_ESM.pdf]

Supplementary Fig 1

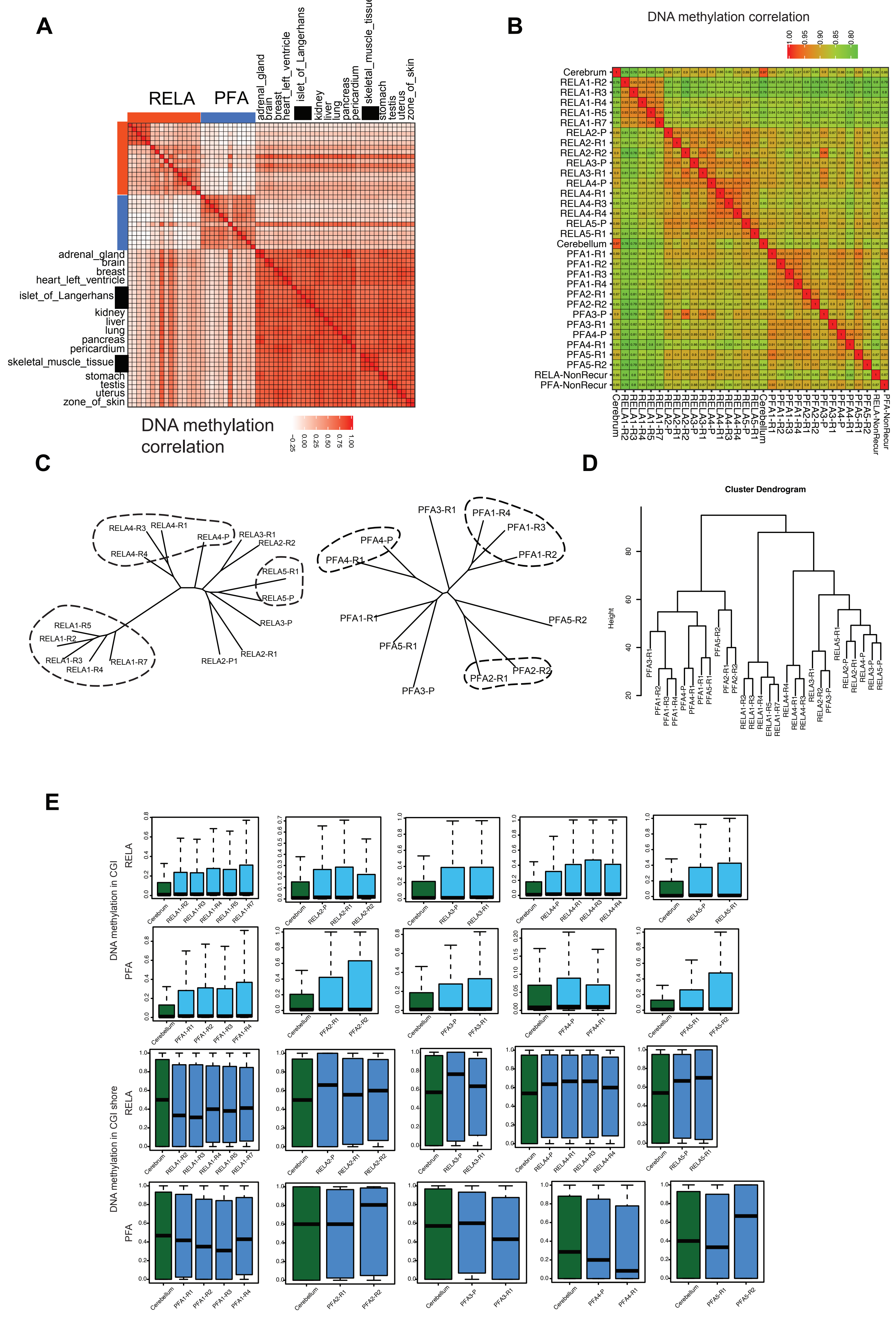

Supplementary Figure 1. DNA methylation profile correlation within samples in this study and among public samples

A. Correlation heatmap of top 20,000 variable CpGs' DNA methylation ratios among RELA/PFA primary/recurrent tumors and normal tissues from ENCODE.

B. Heatmap showing the correlation of DNA methylation profiles of the RELA and PFA tumor samples using normal childhood cerebral and cerebellar tissues as references.

C. Phylogenetic construction of DNA methylation profiles using all RELA patients' samples (left panel) and PFA patients' samples (right panel).

D. Cluster dendrogram for Figure 1C.

E. DNA methylation levels in CpG islands (n=30,000) for each RELA and PFA patient (*upper two panels*); DNA methylation levels in CpG islands shore (n=30,000) for each RELA and PFA patient (*lower two panels)*. Approximately 30,000 CGI shore regions across all samples are included. Each column represents one sample (n=1).

Supplementary Figure 2

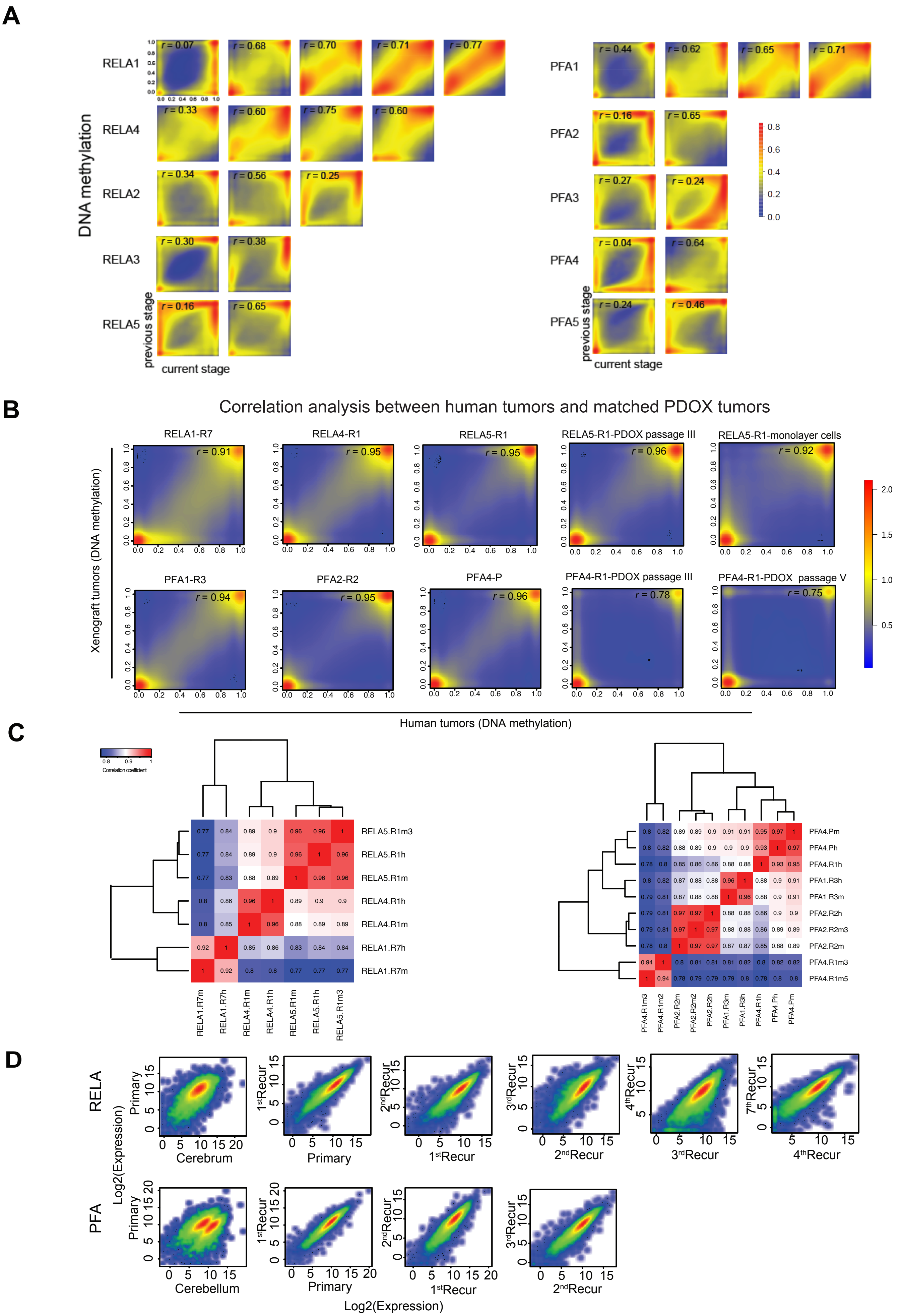

Supplementary Figure 2. Longitudinal analysis of DNA methylation and gene expression profiles along repeated relapses of RELA and PFA ependymomas.

A. Smoothed density scatterplots displaying the gradually convergent pattern of DNA methylation profiles along EPN tumor recur process (across normal cerebrum/cerebellum, RELA/PFA primary tumors, 1st, 2nd and up to 7th recurrent tumors); each dot represents a single CpG; the red color represents high density of dots; the blue color represents low density of dots. x-axis and y-axis represent DNA methylation ratios in corresponding samples. The arrows on the top of the figure showing the EPN tumor recur progress directions.

B. Smoothed density scatterplots showing the correlations of genes expression between tumors from adjacent recurrence stages. Tumors of RELA and PFA were combined; x-axis and y-axis represent gene expression levels.

C. Smoothed density scatterplots showing the correlations of DNA methylation profiles between tumors from patient and tumors from matched PDOX mice models. x-axis and y-axis represent DNA methylation ratios for the overlapped CpGs in patient tumor and tumor from matched PDOX mice, respectively. The sources for PDOX models are labeled on the top of each figure. A monolayer culture derived rom RELA-R1 was also included to compared with the PDOX tumor.

D. Matrix profile showing intersectional correlations of RELA (*left*) and PFA (*right*) tumors between patient tumors (***h***) from primary tumor (***P***) to the first recurrent (***R1***) and up to the 7th recurrent (***R7***) recurrences and PDOX models (**m**) up to passage 3 (***m3***) and 5 (***m5***).

Supplementary Figure 3

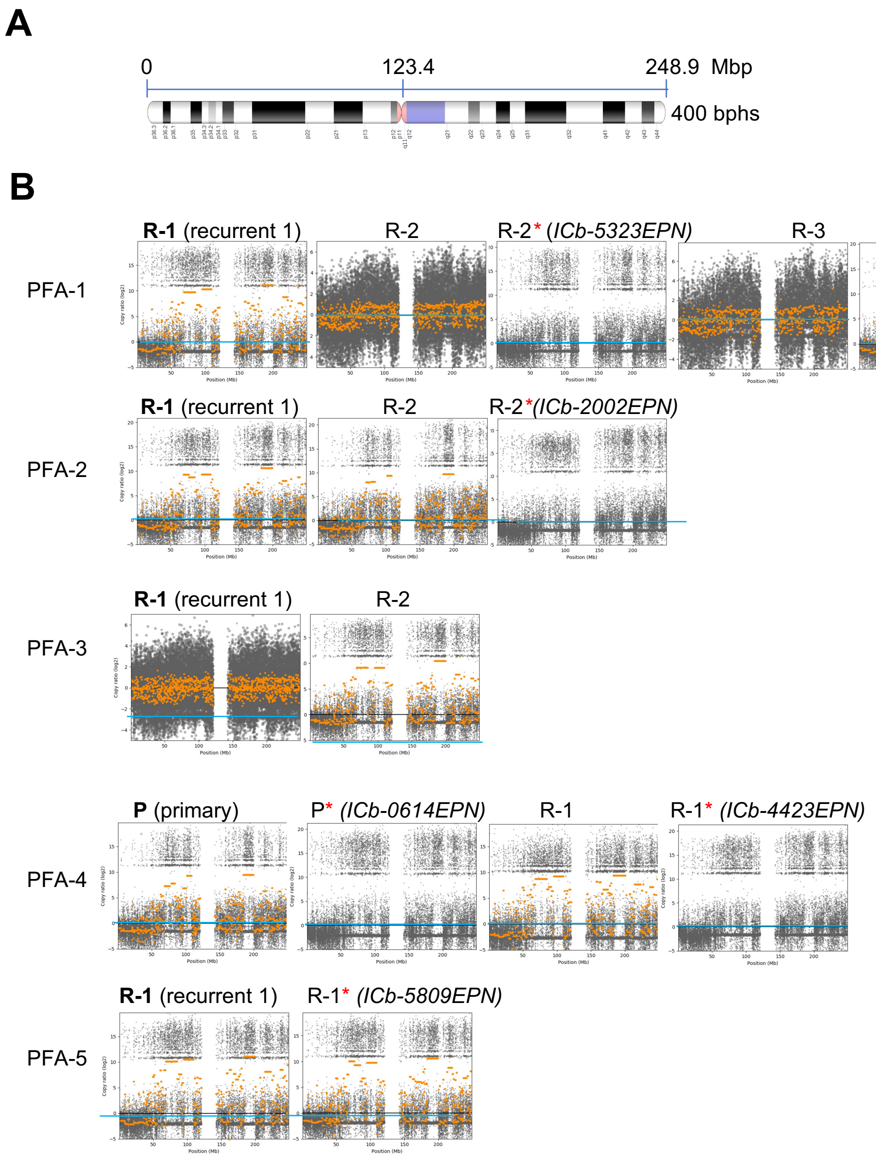

Supplementary Figure 3. CNVkit copy ratios (Chr1) inferred from RRBS of patient and PDOX tumors of EPN.

A. Scheme for Chf1 showing the relative Mb locations on a G-banding (400 band resolution).

B. Plots showing bin levels (log 2) of copy ratios of Chr1 in PFA patient primary (***P***) and recurrent tumors (1~ 4) (***R-1*** to ***R-4***) and along with their PDOX models (*****) and model ID in round brackets. The CNVkits calculate normalized coverage in bin-level (*grey dots*), before removing the systemic bias (such as CG content) use circular binary segmentation (CBS) to infer discreate copy number regions as segments (*yellow dots*). The size of the plotted datapoints is proportional to the weight of each point used in segmentation – a relatively small point indicates a less reliable bin. The dispersion of points around the segmentation line also visually indicates the level of noise or uncertainty.

Supplementary Figure 4

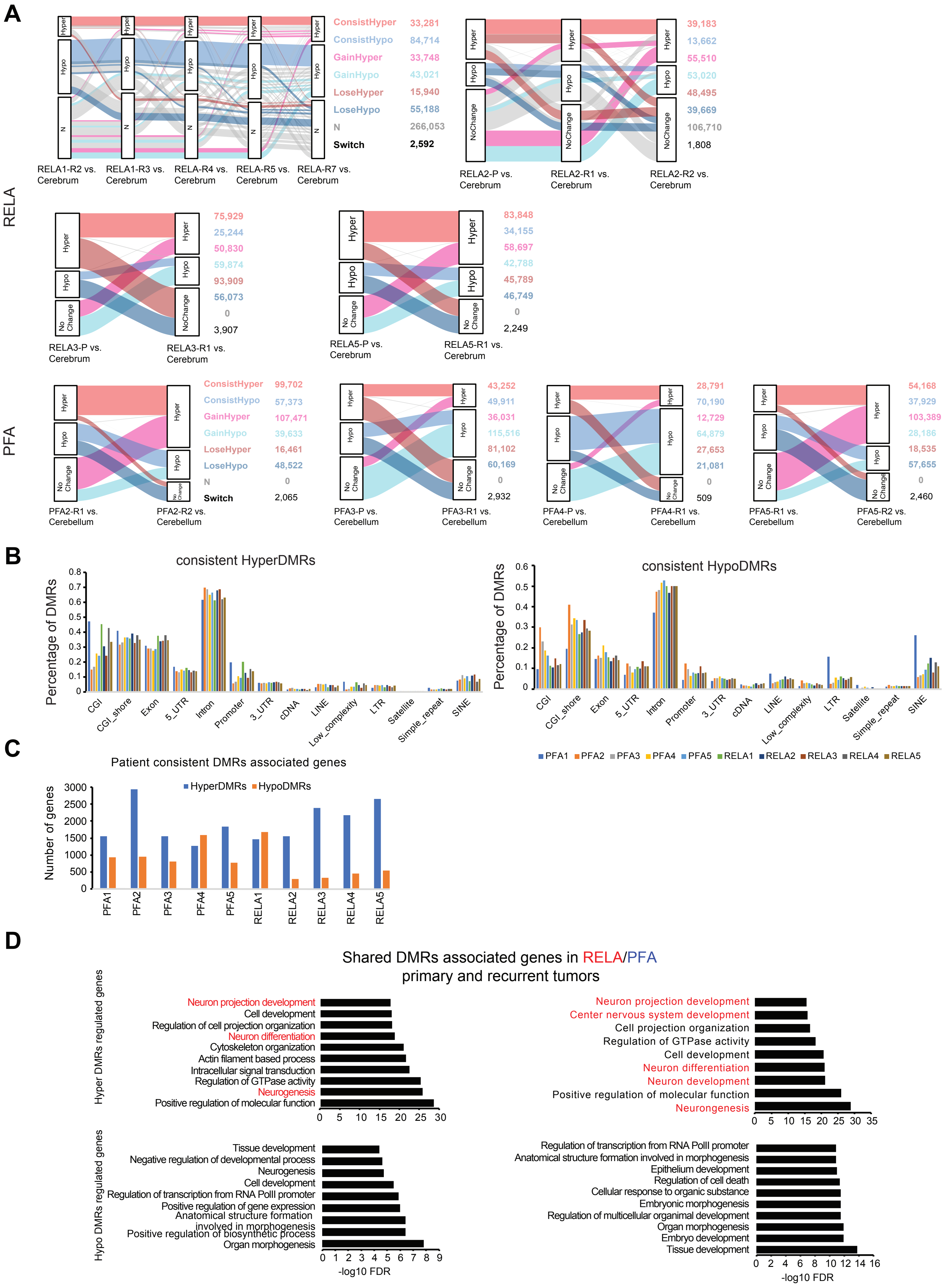

Supplementary Figure 4. Analysis of DNA methylation drivers and their regulated genes and pathways.

A. Alluvia plots showing the dynamic DNA methylation profile changes for the 8 patients (that were not included in the main text) across EPN tumor recur process. Each column represents three status of CpGs (Hyper-, Hypo- and No Change) in the comparison between tumors and normal brain tissues; the three blocks' sizes are scaled to the percentages of DMCs for each status; the bands with 7 different colors (7 categories are defined for the different patterns of CpGs' change) represent the tracking of the CpGs' changes status across recurrent process; the numbers of CpGs for each category are shown on the right side of the each alluvial plot.

B. Histograms showing the relative abundance of consistent HyperDMRs (*upper panel*) and consistent HypoDMRs (*lower pane*) for each patient (labeled as colors) on functional genomic elements.

C. Histogram showing the number of consistent DMRs associated genes for each patient.

D. GSEA analysis of shared DMRs associated genes in RELA (*left panel*) and PFA (*right panel*) patients.

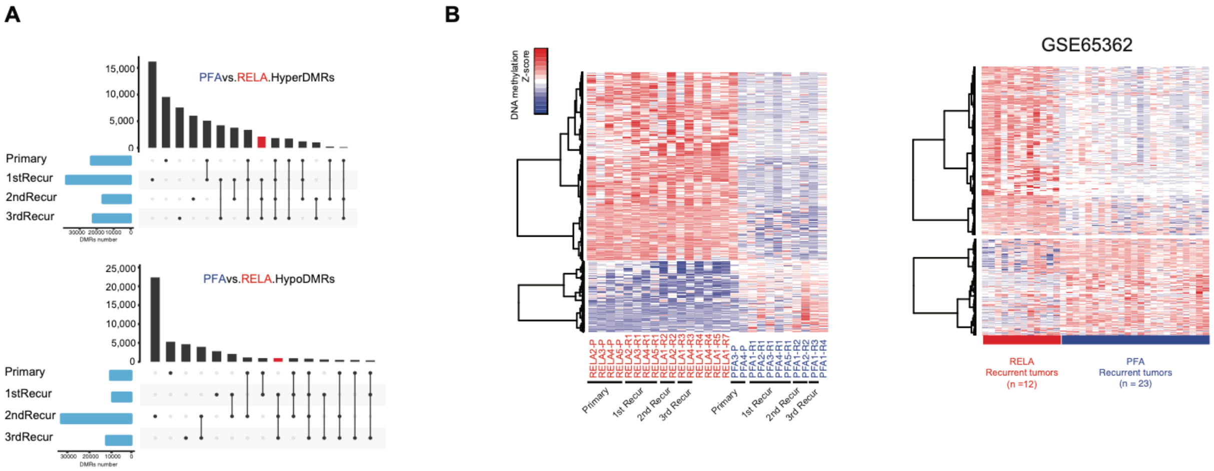

Supplementary Figure 5. Differential DNA methylation profiles between RELA and PFA tumors.

A. UpSetR plot showing the DNA methylation comparisons of primary and recurrent tumors between RELA and PFA EPNs.

B. Heatmaps displaying the DNA methylation levels of CpGs for recurrent samples between RELA and PFA. DNA methylation data of the current (*left panel*) and the same CpGs (of the left panel) for recurrent samples from the public dataset (GSE65362) (*right panel*) are shown.

Supplementary Figure 6

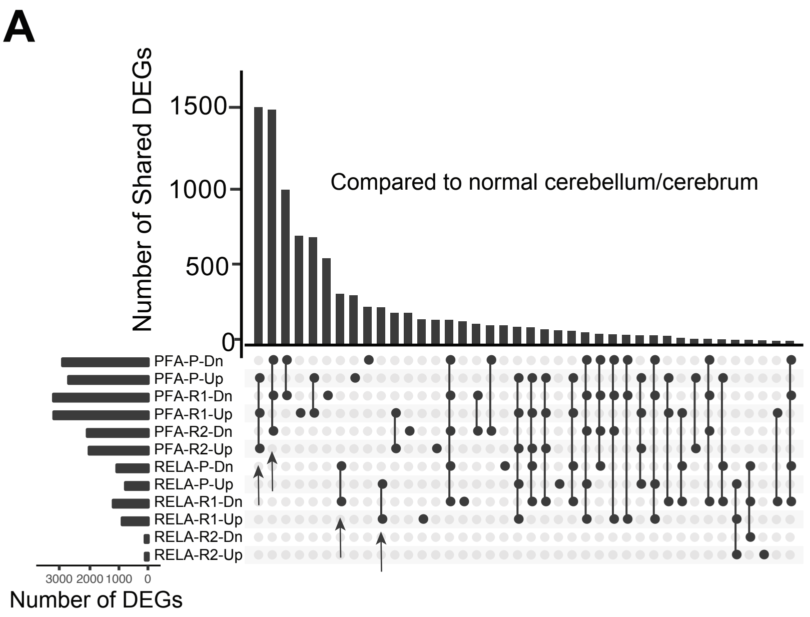

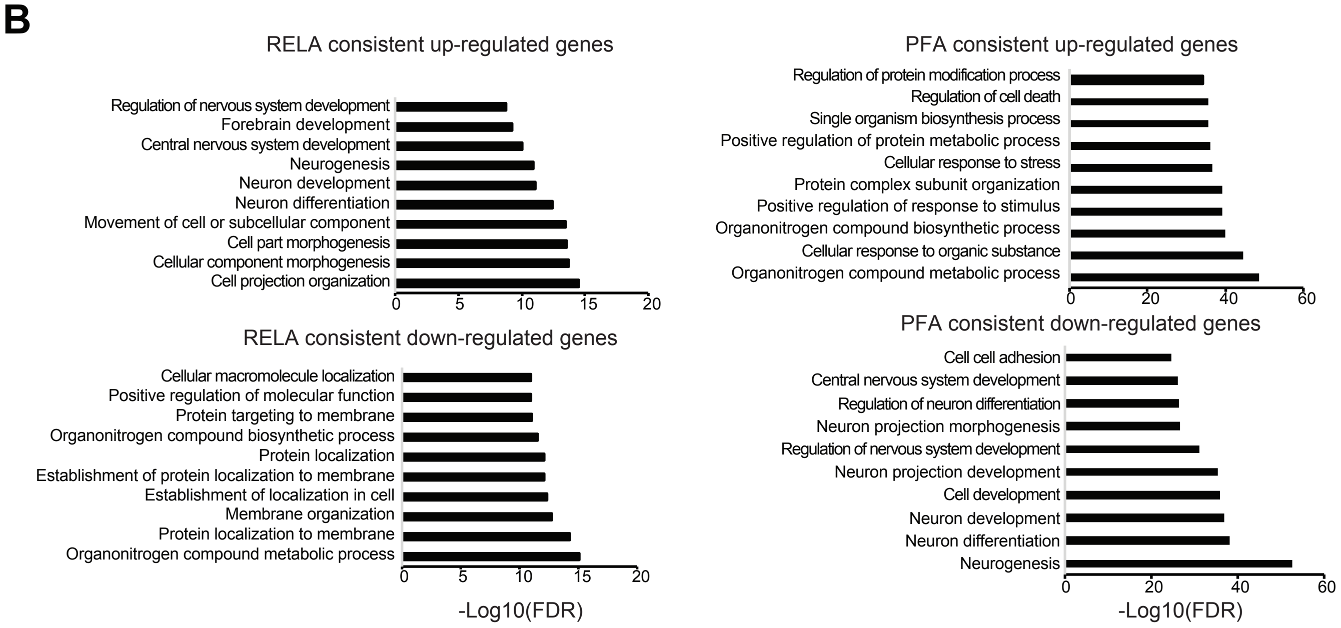

Supplementary Figure 6. Summary of differential expressed genes (DEGs) and the affected signaling pathways during the serial recurrences of childhood ependymoma.

A. UpsetR plot showing the numbers of DEGs by comparing each RELA and PFA tumor tissues to normal cerebral and cerebellar tissues, respectively. The horizonal histogram represents the number of DEGs in each comparison between primary tumor and normal brain tissues; the vertical histogram represents the number of DEGs shared by tumors marked by connected dots. Arrows indicate the genes persisted from the primary to relapsed tumors. (Note of the label: ***P***=primary tumor, ***R1***=first recurrent tumor, ***R2***=Second recurrent tumor, ***Dn***=down regulated, ***Up***=up-regulated).

B. GSEA analysis of RELA/PFA consistent DEGs (up-regulated and down-regulated) among primary tumors, 1st and 2nd recurrent tumors in RELA (*left panel*) and PFA (*right panel*).

Supplementary Figure 7

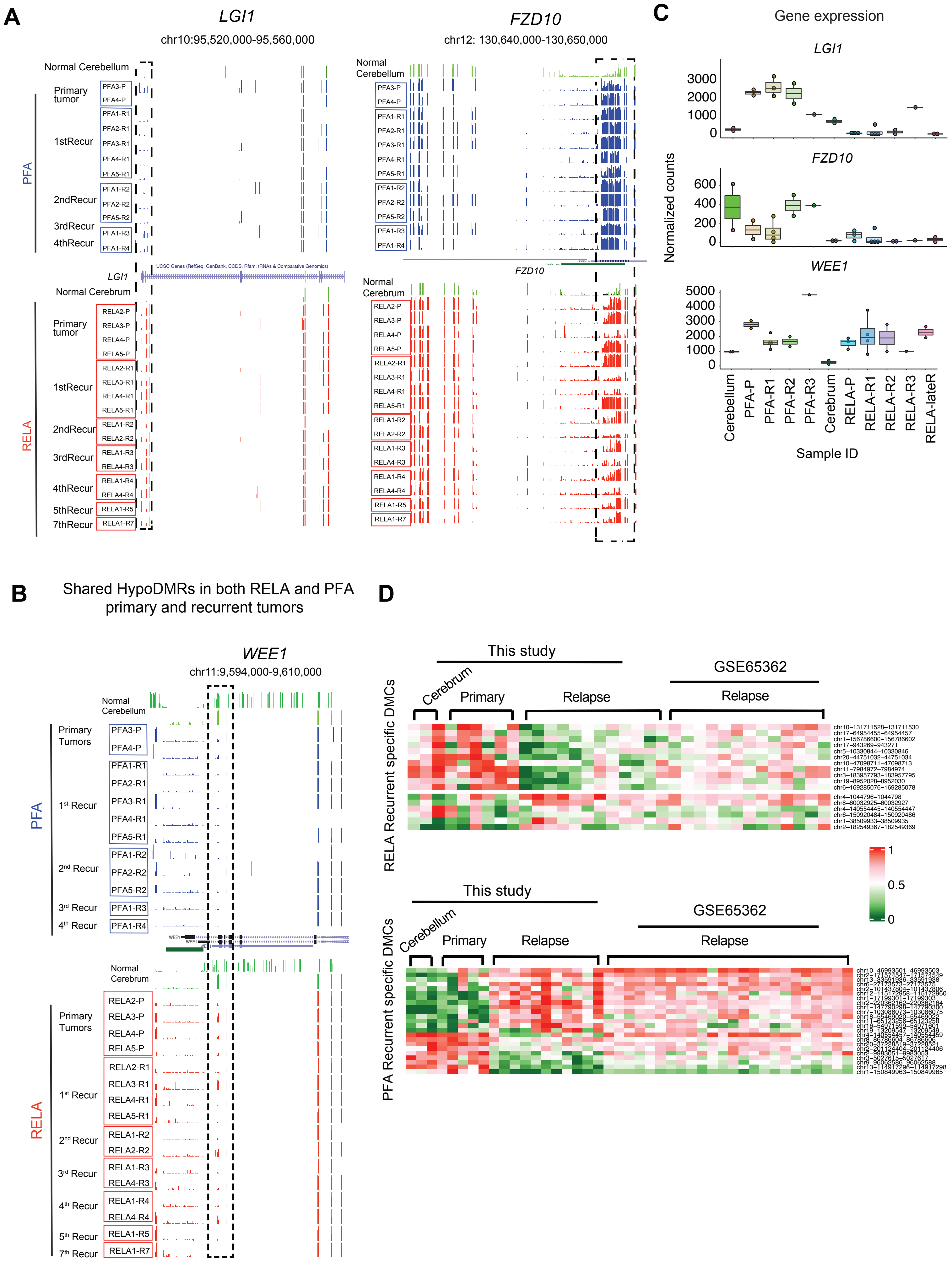

Supplementary Figure 7. UCSC genome browser example views of DNA methylation drivers and target genes

A. Consistent hyperDMR located in *LGI1* promoter region specific to RELA primary and recurrent tumors (*left panel*) and consistent hyperDMR located in *FZD10* proximity region specific in PFA primary and recurrent tumors (*right panel*).

B. HypoDMR region located on *WEE1* gene body occurred in both RELA and PFA primary and recurrent tumors (*left panel*), and hyperDMR region located on proximity of *DLG3* gene in both RELA and PFA primary and recurrent tumors (*right panel*).

C. Boxplots showing the expression levels for *LGI1*, *FZD10*, *WEE1* and *DLG3* genes in RELA and PFA samples (Cerebellum: n=2; PFA-P: n=2; PFA-R1: n=3; PFA-R2:n=2; PFA-R3:n=1; Cerebrum: n=2; RELA-P: n=3; RELA-R1:n=4; RELA-R2:n=2;RELA-R3: n=1; RELA-lateR: n=2). Boxplots indicate median, first and third quartiles (Q1 and Q3), whiskers extend to the furthest values; the uppermost and lowest line indicates the maximum and minimum values, respectively. Each column represents one sample (n=1)

D. Heatmaps showing the levels of a selected set of RELA (upper panel) and PFA (lower panel) recurrent specific CpGs' DNA methylations shared between our data (this study) and a previously published public data (GSE65362)[16].

Supplementary Figure 8

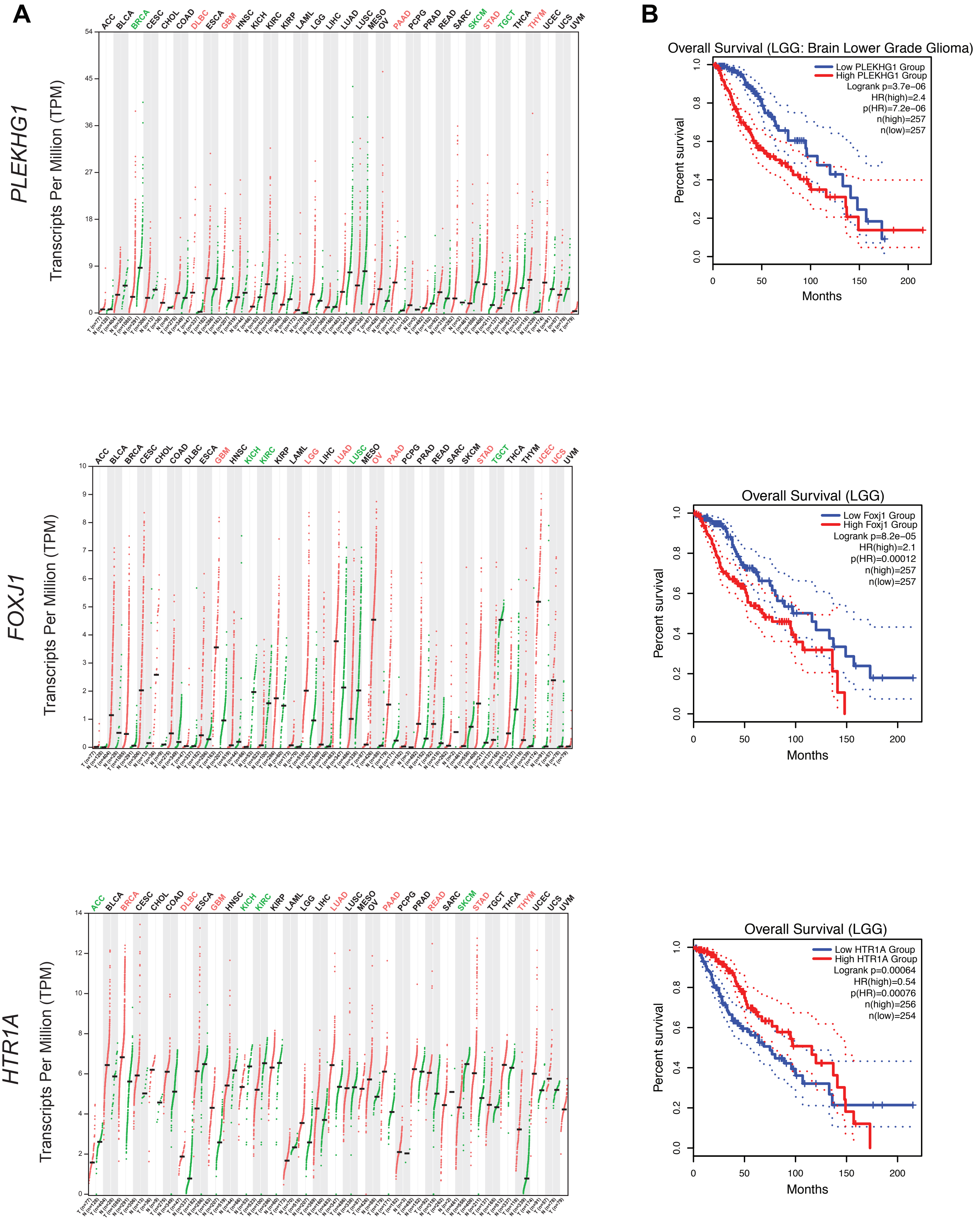

Supplementary Figure 8. Pan-cancer gene expression and survival analysis for DNA methylation drivers associated genes

A. Pan-cancer gene expression analysis for *PLEKHG1*, *FOXJ1* and *HTR1A* genes from TCGA. From the top of each graph, the red and green labeled tumor types indicate that the corresponding gene is significantly up regulated or down regulated in the tumors when compared with the matching normal tissues, respectively. Red dots in the graph indicate tumor (*T*) tissues, while the green dots indicate the normal (*N*) tissues. Median levels of each tumor type were marked with a short black bar.

Abbreviations of diagnosis: ACC (Adrenocortical carcinoma); BLCA (Bladder Urothelial Carcinoma); BRCA (Breast invasive carcinoma); CESC (Cervical squamous cell carcinoma and endocervical adenocarcinoma); CHOL (Cholangio carcinoma); COAD (Colon adenocarcinoma); DLBC (Lymphoid Neoplasm Diffuse Large B-cell Lymphoma); ESCA (Esophageal carcinoma); GBM (Glioblastoma multiforme); HNSC (Head and Neck squamous cell carcinoma); KICH (Kidney Chromophobe); KIRC (Kidney renal clear cell carcinoma); KIRP (Kidney renal papillary cell carcinoma); LAML (Acute Myeloid Leukemia); LGG (Brain Lower Grade Glioma); LIHC (Liver hepatocellular carcinoma); LUAD (Lung adenocarcinoma); LUSC (Lung squamous cell carcinoma); MESO (Mesothelioma); OV (Ovarian serous cystadenocarcinoma); PAAD (Pancreatic adenocarcinoma); PCPG (Pheochromocytoma and Paraganglioma); PRAD (Prostate adenocarcinoma); READ (Rectum adenocarcinoma); SARC (Sarcoma); SKCM (Skin Cutaneous Melanoma); STAD (Stomach adenocarcinoma); TGCT (Testicular Germ Cell Tumors); THCA (Thyroid carcinoma); THYM (Thymoma); UCEC (Uterine Corpus Endometrial Carcinoma); UCS (Uterine Carcinosarcoma); UVM (Uveal Melanoma).

B. Kaplan Meier survival curve analysis for *PLEKHG1*, *FOXJ1* and *HTR1A's* expression using low grade glioma patients' data as an example from TCGA.
