# Supplementary material for: Epigenetic Alterations of Repeated Relapses in Patient-matched Childhood Ependymomas
Source: Nat Commun. 2022 Nov 5;13:6689. doi: 10.1038/s41467-022-34514-z (PMC9637194; doi:10.1038/s41467-022-34514-z)
Supplement: Supplementary file 3 — Description of Additional Supplementary Files [file 41467_2022_34514_MOESM3_ESM.pdf]

File Name: Supplementary Data 1
Description: Summary of DNA methylation sequencing analysis.

File Name: Supplementary Data 2
Description: Correlation of DNA methylation profiles between this study and public data

File Name: Supplementary Data 3
Description: Summary of DMC status.

File Name: Supplementary Data 4
Description: List of potental driver DMCs of recurrence detected in the EPN tumors.

File Name: Supplementary Data 5
Description: Summary of DMRs in RELA and PFA tumors.

File Name: Supplementary Data 6
Description: Candidate driver genes associated with DMRs in RELA tumors.

File Name: Supplementary Data 7
Description: Candidate driver genes associated with DMRs in PFA tumors.

File Name: Supplementary Data 8
Description: List of DMR associated genes that were maintained or lost in the matching PDOX models in RELA and PFA ependymomas.

File Name: Supplementary Data 9
Description: List of DNA methylation booster associated genes that were maintained or lost in the matching PDOX models in RELA and PFA ependymomas.

File Name: Supplementary Data 10
Description: Differentially expressed genes driven by DNA methylation boosters during ependymoma relapses.
