# Supplementary material for: Epigenetic Alterations of Repeated Relapses in Patient-matched Childhood Ependymomas
Source: Nat Commun. 2022 Nov 5;13:6689. doi: 10.1038/s41467-022-34514-z (PMC9637194; doi:10.1038/s41467-022-34514-z)
Supplement: Supplementary file 14 — Reporting Summary [file 41467_2022_34514_MOESM14_ESM.pdf]

nature portfolio

Corresponding author(s): Xiao-Nan Li

Last updated by author(s): Sep 6, 2022

Reporting Summary

Nature Portfolio wishes to improve the reproducibility of the work that we publish. This form provides structure for consistency and transparency in reporting. For further information on Nature Portfolio policies, see our Editorial Policies and the Editorial Policy Checklist.

Statistics

For all statistical analyses, confirm that the following items are present in the figure legend, table legend, main text, or Methods section.

n/a	Confirmed	
☐	☒	The exact sample size (*n*) for each experimental group/condition, given as a discrete number and unit of measurement
☐	☒	A statement on whether measurements were taken from distinct samples or whether the same sample was measured repeatedly
☐	☒	The statistical test(s) used AND whether they are one- or two-sided<br>*Only common tests should be described solely by name; describe more complex techniques in the Methods section.*
☐	☒	A description of all covariates tested
☒	☐	A description of any assumptions or corrections, such as tests of normality and adjustment for multiple comparisons
☐	☒	A full description of the statistical parameters including central tendency (e.g. means) or other basic estimates (e.g. regression coefficient) AND variation (e.g. standard deviation) or associated estimates of uncertainty (e.g. confidence intervals)
☐	☒	For null hypothesis testing, the test statistic (e.g. *F*, *t*, *r*) with confidence intervals, effect sizes, degrees of freedom and *P* value noted<br>*Give P values as exact values whenever suitable.*
☒	☐	For Bayesian analysis, information on the choice of priors and Markov chain Monte Carlo settings
☐	☒	For hierarchical and complex designs, identification of the appropriate level for tests and full reporting of outcomes
☐	☒	Estimates of effect sizes (e.g. Cohen's *d*, Pearson's *r*), indicating how they were calculated

Our web collection on statistics for biologists contains articles on many of the points above.

Software and code

Policy information about availability of computer code

Data collection	Raw RRBS FASTQ files were mapped to NCBI Human Reference Genome Build GRCh37 (hg19) using BSMAP RRBS (v2.9) mode. DNA methylation ratio and differential methylated cytosine (DMCs/DMRs) were analyzed by using MOABS (v1.2.9). CpG sites with five or more reads covered were used for downstream analysis. Bisulfite conversion rates were estimated on the basis of lambda phage genome spike-ins. The bedGraph files including single base pair DNA methylation ratios were transformed to bigwig file format which can be visualized using the UCSC genome browser.
Data analysis	1) DNA methylation heatmaps were plotted using R package https://www.rdocumentation.org/packages/heatmap3/versions/1.1.6/topics/heatmap3 heatmap3 by taking the shared CpGs among all the samples as input. DNA methylation phylogenetics analysis was performed by using R package ape22. To compare multiple groups' DMCs, we merge all the DMCs in all two-group comparisons (union DMC sets). The UpSetR23 package was used to visualize the union of DMC sets among multiple two-group comparisons. To analyze dynamic changes of DMCs among tumor recurrent, we first separate DMCs to three categories (Hyper; Hypo; NoChange) based on adjacent two recurrent stages. Then, by considering four adjacent recurrent stages (P vs. cerebellum; Rl vs. cerebellum; R2 vs. cerebellum and R3 vs. cerebellum), we filtered out those DMCs with hyper/hypo and hypo/hyper switch between any two adjacent stages due to the small numbers. We finally separated DMCs into seven categories: consistent Hyper; consistent Hypo; Gain Hyper; Gain Hypo; Loss Hyper; Loss Hypo and switch (between hyper/hypo and NoChange). R alluvial package (https://www.rdocumentation.org/packages/alluvial/versions/O.l-2/topics/alluvial) was used to visualize the dynamic changes of DMCs along tumor recurrence. Colored density scatterplot of DNA methylation ratios was performed by using R package smoothScatter (https://www.rdocumentation.org/packages/graphics/versions/3.6.l/topics/smoothScatter). GREAT24 was used to predict DMRs' functions. 2) RNAseq: FastQC (http://www.bioinformatics.babraham.ac.uk/projects/fastqc/) was used to do quality checks for raw fastq files. Raw FASTQ files were aligned to NCBI Human Reference Genome Build GRCh37 (hg19) using HISAT216 with default settings. The uniquely mapped reads were used for downstream analysis. HTSeq17 was used to count the reads count mapped in exon regions for each gene. Read counts matrix (row as genes; column as samples) were inputted to DESeq218 to identify differentially expressed

genes (DEGs). We consider genes with FDR<=0.05 and fold change >= 2 folds as DEGs. Principal component analysis of DEGs was performed using R package DESeq2. DEGs' function enrichment was using GSEA 19.

For manuscripts utilizing custom algorithms or software that are central to the research but not yet described in published literature, software must be made available to editors and reviewers. We strongly encourage code deposition in a community repository (e.g. GitHub). See the Nature Portfolio guidelines for submitting code & software for further information.

Data

Policy information about availability of data

All manuscripts must include a data availability statement. This statement should provide the following information, where applicable:

- Accession codes, unique identifiers, or web links for publicly available datasets
- A description of any restrictions on data availability
- For clinical datasets or third party data, please ensure that the statement adheres to our policy

The raw RNAseq and methylation data generated by this study are available from the NCBI under accession number GSE156619 (https://www.ncbi.nlm.nih.gov/geo/query/acc.cgi?acc=GSE156619) The RELA and PFA signature gene list is from public data set GSE64415(https://www.ncbi.nlm.nih.gov/geo/query/acc.cgi?acc=GSE64415)). [16,40,91] Additional RELA and PFA primary tumor WGBS data is from public data set GSE87779 (https://www.ncbi.nlm.nih.gov/geo/query/acc.cgi?acc=GSE87779) [10] and public DNA methylation array data for RELA and PFA relapse samples is from GSE65362 (https://www.ncbi.nlm.nih.gov/geo/query/acc.cgi?acc=GSE65362) [16]. Source data are provided as a Source Data file. The remaining data are available within the Article, Supplementary Information or Source Data file.

Human research participants

Policy information about studies involving human research participants and Sex and Gender in Research.

Reporting on sex and gender	Both genders of patients were included. Animals of both sexes were used in all the tumorigenicity studies.
Population characteristics	All the patients were younger than 21 years (i.e., children) of both genders.
Recruitment	All the patient or families that gave consent to the tumor tissue collection.
Ethics oversight	Institutional Review Board (IRB) of Baylor College of Medicine.

Note that full information on the approval of the study protocol must also be provided in the manuscript.

Field-specific reporting

Please select the one below that is the best fit for your research. If you are not sure, read the appropriate sections before making your selection.

☒ Life sciences ☐ Behavioural & social sciences ☐ Ecological, evolutionary & environmental sciences

For a reference copy of the document with all sections, see nature.com/documents/nr-reporting-summary-flat.pdf

Life sciences study design

All studies must disclose on these points even when the disclosure is negative.

Sample size	Sample size was determined by the tumor tissue availability.
Data exclusions	No data were excluded.
Replication	We used the tumor tissues from the repeated relapses of the same patients; for each subtypes of EPN, we have 5 sets of tumor samples.
Randomization	It is irrelevant to our study.
Blinding	The investigators were blind controlled of molecular subtypes during tumor sample collection.

Reporting for specific materials, systems and methods

We require information from authors about some types of materials, experimental systems and methods used in many studies. Here, indicate whether each material, system or method listed is relevant to your study. If you are not sure if a list item applies to your research, read the appropriate section before selecting a response.

Materials & experimental systems

n/a	Involved in the study
☒	☐ Antibodies
☒	☐ Eukaryotic cell lines
☒	☐ Palaeontology and archaeology
☐	☒ Animals and other organisms
☒	☐ Clinical data
☒	☐ Dual use research of concern

Methods

n/a	Involved in the study
☒	☐ ChIP-seq
☒	☐ Flow cytometry
☒	☐ MRI-based neuroimaging

Animals and other research organisms

Policy information about studies involving animals; ARRIVE guidelines recommended for reporting animal research, and Sex and Gender in Research

Laboratory animals	NOD SCID mice, NOD.129S7(B6)-Ragltm1Mom/J, purchased from Jax Laboratory.
Wild animals	No wide animals were used.
Reporting on sex	Both sexes were used.
Field-collected samples	No filed-collected samples were used.
Ethics oversight	All the experiments were conducted using Institutional Animal Care and Use Committee (IACUC) approved protocols from Baylor College of Medicine or Northwestern University.

Note that full information on the approval of the study protocol must also be provided in the manuscript.
